# Machine learning–based prediction of acute kidney injury after intracerebral hemorrhage: Comparison of multiple model approaches

**DOI:** 10.1097/MD.0000000000046301

**Published:** 2026-05-12

**Authors:** Junzhang Huang, Ningkun Xiao

**Affiliations:** a Department of General Surgery, Lianjiang Traditional Chinese Medicine Hospital, Lianjiang City, Zhanjiang, Guangdong Province, China; b Department of Immunochemistry, Institution of Chemical Engineering, Ural Federal University, Yekaterinburg, Russia; c Department of Psychology, Laboratory for Brain and Neurocognitive Development, Institution of Humanities, Ural Federal University, Yekaterinburg, Russia.

**Keywords:** acute kidney injury, bibliometric analysis, intracerebral hemorrhage, machine learning, mediation analysis

## Abstract

To identify key risk factors for acute kidney injury (AKI) in patients with intracerebral hemorrhage (ICH) using bibliometric analysis and machine learning, and to explore the mediating role of hemoglobin (Hb) in the association between hypertension and AKI. A bibliometric analysis of English-language publications from 2014 to 2024 was conducted to evaluate research trends and thematic clusters related to ICH and AKI. Clinical data from 944 ICH patients in the Medical Information Mart for Intensive Care-IV (MIMIC-IV) database were analyzed, including 46 patients who developed AKI. A total of 120 machine learning model combinations were developed and compared using area under the receiver operating characteristic curve (AUC). SHapley Additive exPlanations (SHAP) and clustering heatmaps were employed to interpret feature importance and patient-level heterogeneity. Mediation analysis was used to assess the indirect effect of Hb on the relationship between hypertension and AKI. A total of 122 relevant publications were identified, revealing a growing academic focus on integrated neurocritical care and the emergence of stable interdisciplinary research clusters. Among the machine learning models, the Random Forest (RF) + NaiveBayes + Tree ensemble achieved the highest predictive performance (AUC = 0.764). SHAP analysis identified platelet count (PLT) as the most influential predictor of AKI risk. Clustering analysis revealed substantial heterogeneity in feature contributions across patient subgroups. Mediation analysis confirmed that Hb significantly and negatively mediated the effect of hypertension on AKI occurrence (*P* < .05). The RF + NaiveBayes + Tree ensemble demonstrated superior predictive power for early AKI risk identification. SHAP analysis highlighted PLT as a key predictor, with notable inter-individual variability in risk profiles. The observed mediating effect of Hb between hypertension and AKI offers exploratory mechanistic insights that warrant further validation. While these findings contribute to the understanding of ICH-associated AKI, the current model remains exploratory and is not yet ready for direct clinical implementation. Future studies with larger, multicenter cohorts are needed to refine the model and assess its clinical utility.

## 1. Introduction

Intracerebral hemorrhage (ICH) is one of the most devastating acute neurological conditions, characterized by high incidence, mortality, and long-term disability rates.^[1–3]^ Despite notable advances in critical care and treatment strategies, the burden of post-ICH complications remains substantial and continues to challenge clinical management. Among these, acute kidney injury (AKI) stands out as a frequent and serious complication, markedly increasing hospital length of stay, healthcare costs, and mortality risk.^[4–7]^ AKI has thus emerged as a critical determinant of prognosis and therapeutic decision-making in patients with ICH.

Current research at the intersection of ICH and AKI remains fragmented and underdeveloped. The field is hindered by limited predictive tools, insufficient integration of multidisciplinary approaches, and a lack of systematic investigation into the underlying mechanisms linking ICH and renal dysfunction. Conventional statistical models often fall short in capturing the complexity and nonlinearity inherent in high-dimensional clinical datasets, impeding the identification of nuanced risk patterns and latent interactions.^[8,9]^

To bridge these gaps, our study begins with a bibliometric analysis not merely as a descriptive exercise but as a data-driven strategy to map the intellectual structure and evolution of ICH–AKI research. This step allows us to identify dominant research themes, emerging hotspots, and underexplored areas, thereby providing a conceptual foundation for hypothesis generation and model design. For instance, bibliometric keyword clustering revealed recurring attention to hemodynamic instability, inflammatory responses, and renal perfusion, which guided our feature selection and informed the inclusion of hypertension- and hemoglobin-related variables in the subsequent modeling stage.

However, despite recent progress in machine learning–based prediction of AKI, few studies have specifically focused on the neurocritical population of ICH patients, and even fewer have incorporated explainable artificial intelligence frameworks to elucidate model reasoning or validate findings across large, real-world cohorts. This lack of explainable and externally validated predictive models represents a key research gap, limiting the clinical applicability and interpretability of AI-assisted risk assessment in ICH-related AKI.

To address these gaps, this study first conducts a comprehensive bibliometric analysis of the global literature on ICH-associated AKI from 2014 to 2024. Through an integrative approach involving publication trend analysis, keyword co-occurrence mapping, and topic clustering, we delineate the evolving research landscape, highlight major knowledge domains, and identify critical limitations in the existing body of work. This mapping provides a foundational overview to guide future research priorities and foster interdisciplinary dialogue.

Based on these insights, we use the Medical Information Mart for Intensive Care-IV (MIMIC-IV) database to establish multiple machine learning algorithm combination models and compare the advantages and disadvantages of these machine learning algorithm combinations. We applied Shapley additive explans (SHAP) to improve the interpretability of the model and reveal the relative contributions of clinical variables. Furthermore, we incorporate causal mediation analysis to explore a potential mechanistic pathway linking hypertension and AKI via hemoglobin levels. This dual modeling and mechanistic approach aims not only to improve predictive performance but also to shed light on the complex pathophysiological interplay between the central nervous system and renal function.

Overall, our study provides an integrated framework that combines bibliometric mapping, predictive analytics, and mechanistic exploration to advance the understanding and management of AKI following ICH. These findings are intended to support early identification, targeted intervention, and translational research in this high-risk patient population.

## 2. Methods

### 2.1. Study design

**Figure 1.**
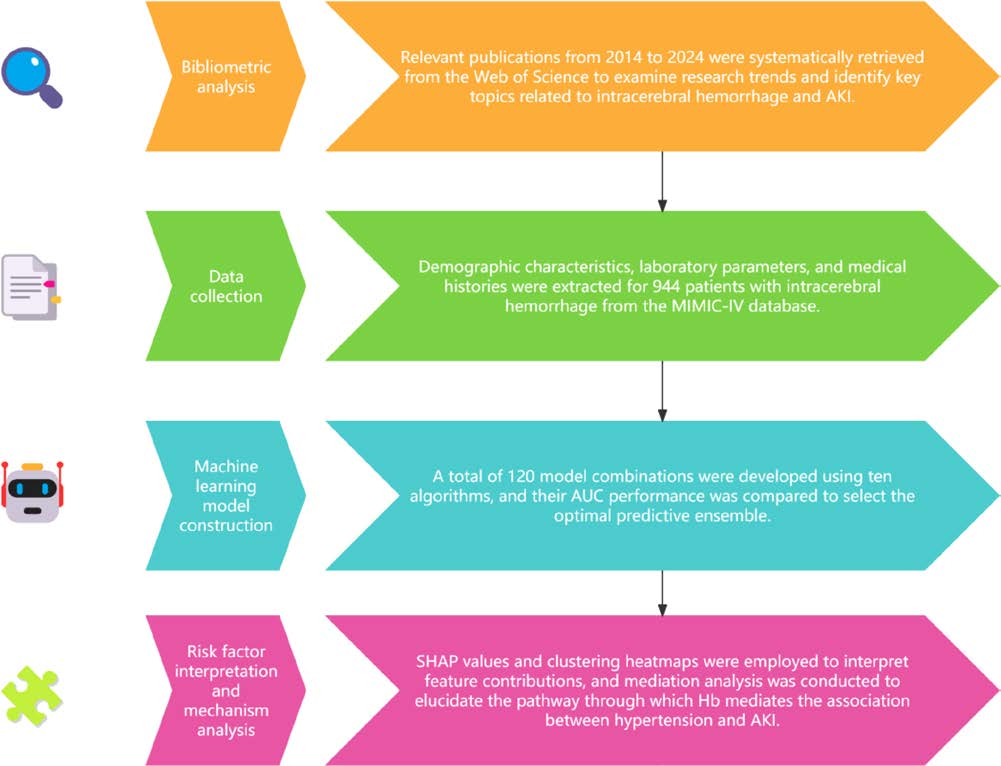
Research process design diagram.

A comprehensive overview of the study design is presented in Figure 1, which outlines the integrated workflow encompassing 4 major components: a bibliometric analysis to map research trends and thematic evolution in the field of ICH complicated by AKI; extraction of demographic, laboratory, and clinical data from the MIMIC-IV database; development and performance evaluation of multiple machine learning models for AKI risk prediction; and interpretation of model results using SHAP for feature importance, followed by mediation analysis to explore mechanistic pathways.

### 2.2. Bibliometric analysis

We conducted a literature search via the Web of Science Core Collection database, covering the period from January 1, 2014, to December 31, 2024. The search strategy combined terms related to cerebral hemorrhage and AKI, including: “intracerebral hemorrhage,” “cerebral hemorrhage,” “brain hemorrhage,” “ICH,” “acute kidney injury,” “AKI,” and “acute renal failure.” The search was restricted to English-language publications and limited to original research articles and reviews. The bibliometric analysis included evaluation of annual publication trends, keyword co-occurrence networks, and topic clustering using VOSviewer and R-based visualization tools.

### 2.3. Study population

Clinical data were extracted from the publicly available MIMIC-IV (v3.0) database, a large, de-identified electronic health record resource developed by the Massachusetts Institute of Technology in collaboration with Beth Israel Deaconess Medical Center.^[10]^ The database contains detailed information on intensive care unit (ICU) admissions, including vital signs, laboratory results, diagnoses, procedures, and treatment records.

For this study, we included adult patients (aged 18–100 years) with a diagnosis of ICH who had ICU stays of at least 72 hours. To ensure consistency, only the first ICU admission was considered for patients with multiple admissions. A total of 944 eligible patients were identified, of whom 46 developed AKI during their ICU stay (AKI group), while the remaining 898 formed the non-AKI group.

### 2.4. Data collection

We retrieved a wide range of variables from the database, including demographic data: age, gender, weight, and height; laboratory variables: complete blood count, serum creatinine, hemoglobin, and other routine clinical tests; clinical history and comorbidities: hypertension, diabetes mellitus, myocardial infarction, and other relevant conditions. Before statistical modeling, all data underwent quality control procedures, including missing data screening, multiple imputation, outlier detection, and normalization. Before statistical modeling, all data underwent quality control procedures, including missing data screening, mean imputation, outlier detection, and normalization.

## 3. Statistical analysis

To construct predictive models for AKI risk, we implemented a suite of supervised machine learning algorithms, including Logistic Regression, Random Forest (RF), Least Absolute Shrinkage and Selection Operator (LASSO), Naive Bayes, K-Nearest Neighbors (KNN), Decision Tree, Support Vector Machine (SVM), Neural Network (NN), eXtreme Gradient Boosting (XGBoost), and Light Gradient Boosting Machine (LightGBM). In total, 120 model combinations were tested through hyperparameter tuning and cross-validation procedures. Predictive performance was evaluated using the area under the receiver operating characteristic curve (AUC), and models were ranked accordingly.

To enhance model interpretability and identify the most influential predictors, we applied the SHAP method. SHAP summary plots and clustered heatmaps were used to visualize the direction and magnitude of each variable’s impact on model outputs across patient groups.

### 3.1. Mediation analysis

To further elucidate potential mechanistic pathways, we conducted a causal mediation analysis to examine whether hemoglobin levels mediate the relationship between hypertension and AKI occurrence in ICH patients. Mediation modeling was performed using standard regression-based approaches with bootstrap estimation for significance testing.

All statistical analyses and visualizations were conducted using R software (version 4.2.0) and complementary R-based online analysis platforms.

## 4. Results

### 4.1. Bibliometric analysis

**Figure 2.**
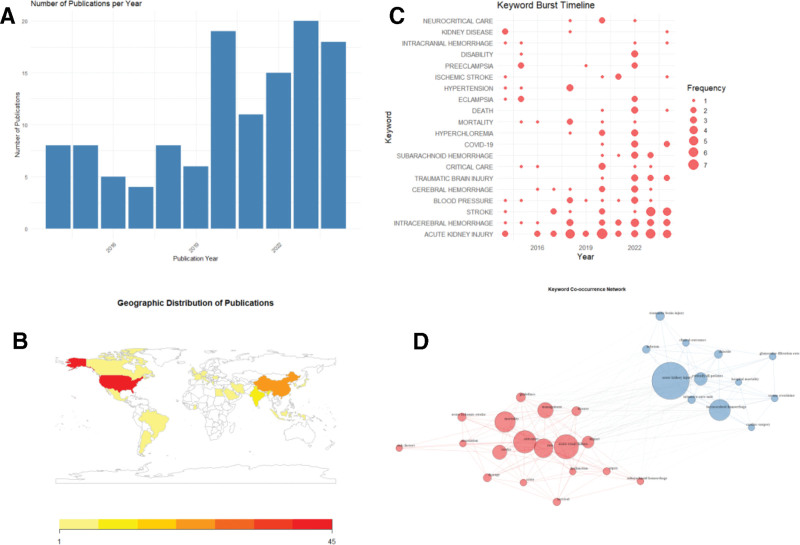
Bibliometric analysis. (A) Bar chart. (B) Geographic heatmap. (C) Keyword burst timeline. (D) Keyword co-occurrence network.

A total of 122 publications related to cerebral hemorrhage complicated by AKI were retrieved from the Web of Science database. Figure 2A illustrates the annual publication trends from 2014 to 2024. The number of publications displays a clear upward trajectory, reflecting the growing academic interest in this interdisciplinary topic. During the initial phase (2014–2019), annual publication counts remained low and exhibited considerable fluctuation, suggesting that the field was still in its formative stage. A marked increase in output began in 2020, likely driven by heightened clinical awareness of multi-organ complications and increased integration of neurological and renal research. The number of publications peaked in 2023, highlighting the emergence of this topic as a focal point in translational and critical care medicine. This overall upward trend underscores the increasing recognition of the clinical and research significance of AKI in the context of intracerebral hemorrhage.

Figure 2B shows the global research contribution map, based on the affiliation of the first author’s country, with dark shading indicating a higher number of publications. The United States is leading in this field, contributing the largest amount of research and becoming a key driving force for innovation and cooperation. The research output of China and India has also significantly increased, indicating a continuous growth in research investment in these regions. The stable contributions of European countries, as well as Canada and Australia, reflect the breadth of international participation. Overall, these findings reveal a research landscape led by the United States and involving global participation, implying vast potential for international cooperation and knowledge exchange.

Figure 2C shows a temporal keyword burst chart, reflecting fluctuations in keyword citation frequency. Terms such as “acute kidney injury,” “cerebral hemorrhage,” and “stroke” have shown significant bursts in recent years, indicating a shift in literature topics from isolated organ pathophysiology to multi-organ interactions, systemic complications, and comprehensive management methods.

Figure 2D presents a co-occurrence network of keywords, displaying multiple stable thematic sub domains, including terms such as “acute kidney injury” and “cerebral hemorrhage,” involving research on clinical outcomes and prognosis; Another cluster focuses on terms such as “acute renal failure,” “sepsis,” and “mortality,” involving research on critical care and systemic complications. The co-occurrence of keywords reveals the close connections between neurology, nephrology, and critical care medicine, reflecting interdisciplinary research trends. The maturity of these clusters reflects the transformation of the field towards precision medicine and personalized intervention strategies, providing new directions for the management of AKI related to acute cerebral hemorrhage.

### 4.2. Machine learning models

A total of 944 cerebral hemorrhage patients were included in the study, with 46 diagnosed with AKI and 898 in the non-AKI group. The baseline characteristics of both groups are summarized in Table 1. The mean age of patients in the AKI group was 64.1 ± 16.9 years, compared to 68.0 ± 15.6 years in the non-AKI group. Notably, patients in the AKI group had a significantly higher mean body weight (88.7 ± 26.0 kg) than those in the non-AKI group (79.9 ± 21.9 kg).

**Table 1:** Baseline characteristic.

Characteristic	AKI, n = 46	Non-AKI, n = 898
Age, yr, Mean ± SD	64.1 ± 16.9	68.0 ± 15.6
Weight, kg, Mean ± SD	88.7 ± 26.0	79.9 ± 21.9
Height, cm, Mean ± SD	170.9 ± 10.4	168.4 ± 7.7
WBC, K/uL, Mean ± SD	12.7 ± 6.5	12.2 ± 15.0
RBC, m/uL, Mean ± SD	3.6 ± 0.9	4.1 ± 0.7
Hb, g/dL, Mean ± SD	10.8 ± 2.6	12.2 ± 2.1
PLT, K/uL, Mean ± SD	172.0 ± 93.5	219.1 ± 80.8
ANC, K/uL, Mean ± SD	9.7 ± 4.7	9.7 ± 3.8
ALC, K/uL, Mean ± SD	1.6 ± 0.9	1.9 ± 7.4
Eosinophil, %, Mean ± SD	1.7 ± 2.1	1.2 ± 1.1
Basophil, %, Mean ± SD	0.3 ± 0.2	0.4 ± 0.2
Gender, n (%)		
Female	12 (26.1%)	414 (46.1%)
Male	34 (73.9%)	484 (53.9%)
Language, n (%)		
English	40 (87.0%)	772 (86.0%)
Spanish	0 (0.0%)	40 (4.5%)
Portuguese	0 (0.0%)	10 (1.1%)
Kabuverdianu	0 (0.0%)	11 (1.2%)
Chinese	1 (2.2%)	18 (2.0%)
Haitian	3 (6.5%)	15 (1.7%)
Khmer	0 (0.0%)	4 (0.4%)
Arabic	0 (0.0%)	3 (0.3%)
Other	0 (0.0%)	3 (0.3%)
Vietnamese	0 (0.0%)	6 (0.7%)
Korean	1 (2.2%)	2 (0.2%)
Russian	1 (2.2%)	9 (1.0%)
Persian	0 (0.0%)	2 (0.2%)
Greek	0 (0.0%)	1 (0.1%)
Italian	0 (0.0%)	1 (0.1%)
Armenian	0 (0.0%)	1 (0.1%)
Marital status, n (%)		
Married	32 (69.6%)	579 (64.5%)
Single	9 (19.6%)	178 (19.8%)
Divorced	2 (4.3%)	59 (6.6%)
Widowed	3 (6.5%)	82 (9.1%)
Hypertension, n (%)		
No	27 (58.7%)	349 (38.9%)
Yes	19 (41.3%)	549 (61.1%)
Diabetes, n (%)		
No	29 (63.0%)	666 (74.2%)
Yes	17 (37.0%)	232 (25.8%)
HF, n (%)		
No	30 (65.2%)	777 (86.5%)
Yes	16 (34.8%)	121 (13.5%)
MI, n (%)		
No	39 (84.8%)	848 (94.4%)
Yes	7 (15.2%)	50 (5.6%)
CA, n (%)		
No	41 (89.1%)	762 (84.9%)
Yes	5 (10.9%)	136 (15.1%)
Pneumonia, n (%)		
Yes	16 (34.8%)	257 (28.6%)
No	30 (65.2%)	641 (71.4%)
HLP, n (%)		
No	26 (56.5%)	514 (57.2%)
Yes	20 (43.5%)	384 (42.8%)

AKI = acute kidney injury, ALC = absolute lymphocyte count, ANC = absolute neutrophil count, CA = cancer, Hb = hemoglobin, HF = heart failure, HLP = hyperlipidemia, MI = myocardial infarction, PLT = platelet count, RBC = red blood cell count, WBC = white blood cell count.

**Figure 3.**
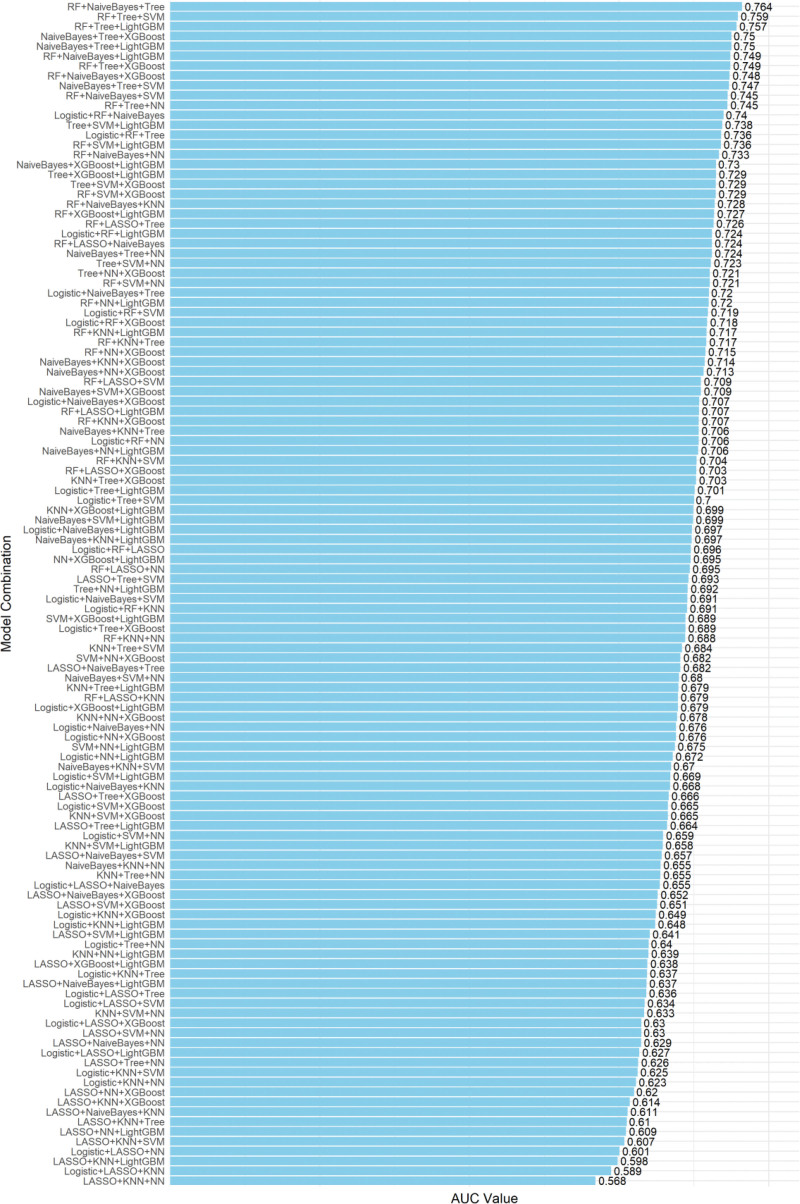
AUC of machine learning algorithm models. Machine learning algorithms: Logistic, RF, LASSO, NaiveBayes, KNN, Tree, SVM, NN, XGBoost, LightGBM. AUC = area under the curve, KNN = K-nearest neighbors, LightGBM = light gradient boosting machine, RF = random forest, SVM = support vector machine, XGBoost = extreme gradient boosting.

We developed and evaluated various machine learning models using the clinical features in Table 1 to predict AKI in patients with cerebral hemorrhage. As shown in Figure 3, the AUC values of 120 combinations of machine learning algorithms were calculated and sorted in descending order. The horizontal bar chart intuitively emphasizes the differences in AUC performance between different combinations of machine learning algorithms. The best performing ensemble consisting of RF + NaiveBayes + Tree achieved the highest AUC value, with an AUC value of 0.764, indicating excellent discriminative ability in identifying AKI cases.

### 4.3. SHAP analysis and clustering heatmap

**Figure 4.**
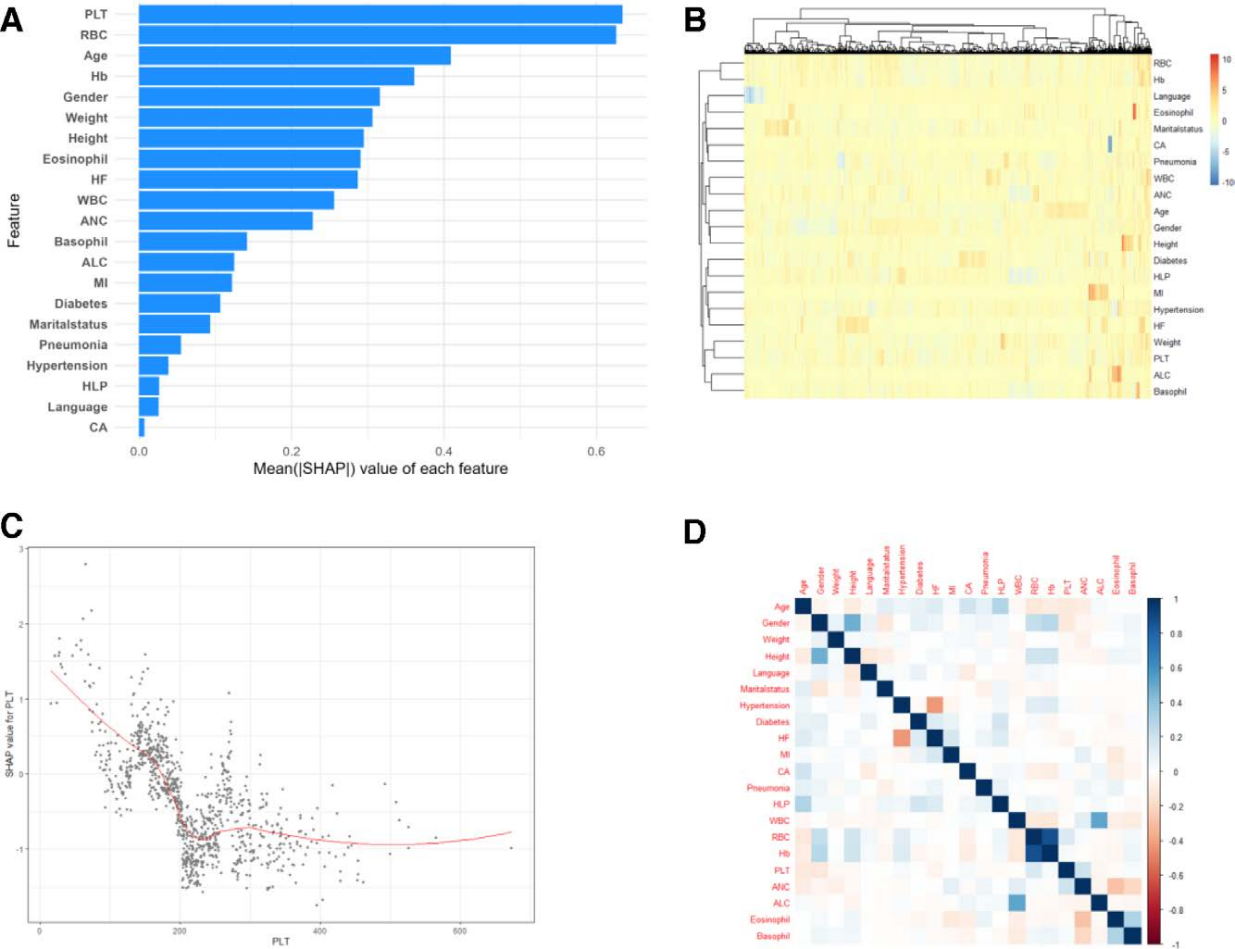
Importance and heterogeneity distribution of risk characteristics. (A) Bar plot. (B) Dependence plot. (C) Clustering heatmap. (D) Matrix heatmap.

To identify key predictors of AKI and interpret model output, we employed the SHAP method. As presented in Figure 4A, the SHAP summary plot ranked PLT as the most important predictor, contributing the highest mean SHAP value. This finding highlights the critical role of hemodynamic and hematologic variables in AKI risk stratification among ICH patients.

To further investigate the impact of blood PLT on the risk of AKI, a SHAP dependent graph (Fig. 4B) was constructed. The graph shows the nonlinear relationship between platelet count and SHAP values. Specifically, lower platelet counts are associated with higher SHAP values, indicating an increased risk of predicted AKI. This nonlinear pattern reveals potential threshold effects and emphasizes the importance of platelet count as a dynamic and sensitive predictor.

Further analysis using a SHAP value clustering heatmap revealed distinct distribution patterns of feature contributions across different patients (Fig. 4C). There was marked heterogeneity in the direction and magnitude of feature contributions at the individual level, particularly for indicators such as RBC and Hb, which showed clusters of abnormal SHAP values. Moreover, variables like Hypertension and MI exhibited abnormally high or low SHAP values in certain patients, suggesting that these features may serve as sensitive risk factors for AKI in specific subgroups.

A correlation matrix heatmap was employed to explore inter-variable relationships among all predictors (Fig. 4D). The heat map results show a moderate correlation between some hematological indicators, such as RBC and Hb. Most clinical variables exhibited weak correlations with each other, suggesting relative independence in their contributions to the model. This pattern supports the multivariate robustness of the SHAP-based interpretation by minimizing potential multicollinearity bias among predictors.

### 4.4. Mediation analysis

**Figure 5.**
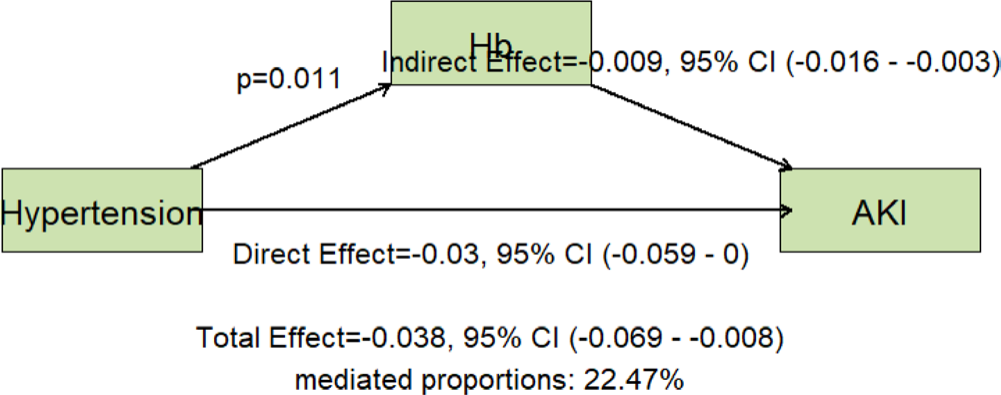
Mediating effect pathway.

To explore the potential mechanistic pathway through which hypertension contributes to AKI in patients with ICH, a causal mediation analysis was conducted. The analysis revealed that Hb played a significant mediating role ((*P* < .05). As shown in Figure 5, Hb exhibited a significant negative indirect effect on AKI risk, with an effect estimate of − 0.009 and 95% confidence interval (CI): −0.016 to −0.003. This suggests that hypertension may increase AKI susceptibility by reducing Hb levels, thereby impairing oxygen delivery and renal perfusion in critically ill patients.

These findings offer new insights into the neuro-renal axis and suggest that maintaining adequate Hb levels may represent a modifiable intervention target to mitigate AKI risk among hypertensive patients with ICH.

## 5. Discussion

The bibliometric analysis in our study reveals a clear upward trajectory in academic interest at the intersection of ICH and AKI over the past decade. From 2014 to 2024, there has been a steady increase in publications, with a particularly sharp rise after 2020. This trend likely reflects the growing recognition that AKI is a critical complication in neurocritical care patients and the broader shift towards multi-organ management in critical illness.^[11,12]^ In fact, some literature emphasizes that a portion of neurological intensive care patients may experience AKI, which in turn exacerbates neurological damage through metabolic and inflammatory pathways.^[13–15]^

These insights highlight a paradigm shift: neurological intensive care is increasingly adopting a comprehensive, multidisciplinary approach that incorporates kidney protection into the management of neurological intensive care patients. The surge in publications coinciding with advances in big data and artificial intelligence likely indicates that researchers are leveraging large databases and machine learning techniques to predict adverse outcomes and personalize patient care.^[15–20]^ This aligns with global precision medicine efforts to account for systemic organ crosstalk—moving beyond a siloed focus on the brain alone toward comprehensive risk assessment across organ systems.

Geographically, the United States has dominated research output in this domain, likely owing to robust critical care research infrastructure and access to extensive datasets (such as MIMIC-IV and the Nationwide Inpatient Sample). The strong U.S. contribution may also reflect multidisciplinary collaborations between neurologists, nephrologists, and data scientists in major academic centers. At the same time, significant contributions are emerging from Asia (notably China and India), indicating expanding global interest. The involvement of diverse regions is encouraging for future international collaborations, which could standardize definitions and management strategies for AKI in neurocritical care across different healthcare systems. In our analysis, European and Oceanian researchers also showed active participation, reinforcing that the clinical challenge of ICH-associated AKI is universally recognized. This worldwide engagement sets the stage for consensus-building and large multicenter studies that can validate findings across populations.

**Figure 6.**
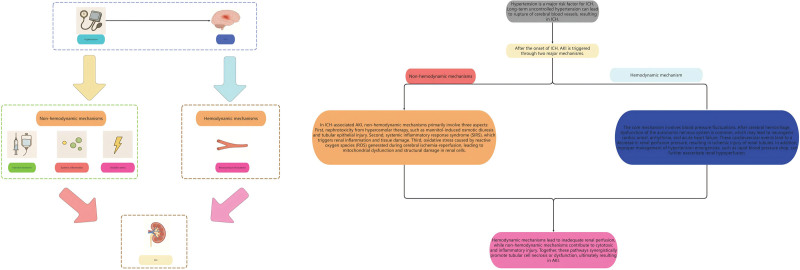
Mechanism diagram.

The content analysis of keywords and co-citation networks illustrates a clear thematic evolution in the literature over time. Early studies in this field focused on epidemiology and definitions—for example, establishing how frequently AKI occurs after hemorrhagic stroke and the variability in diagnostic criteria. By contrast, recent research has shifted toward elucidating pathophysiological mechanisms and complex inter-organ interactions. Key mechanistic themes that have gained prominence include hemodynamic instability (e.g., blood pressure fluctuations and neurogenic cardiac stunning), neurohumoral activation, systemic inflammation, and metabolic disturbances.^[21–27]^ Meanwhile, inflammatory cytokines and oxidative stress caused by ICH can directly damage kidney cells, and common interventions such as hypertonic treatment with mannitol can cause additional iatrogenic stress to the kidneys. This convergence of hemodynamic and nonhemodynamic factors forms a complex “brain–kidney axis” in ICH.^[28]^ ICH triggers both hemodynamic mechanisms (e.g., neurogenic cardiac stunning with reduced renal perfusion) and nonhemodynamic mechanisms (neurohumoral activation, inflammatory and oxidative injury, plus iatrogenic factors like nephrotoxic drugs), which together contribute to AKI (Fig. 6).

Our study contributes to the emerging literature on predictive analytics in neurocritical care by evaluating a broad array of machine learning models for AKI prediction. Notably, out of 120 model combinations tested, the best performance was achieved by an ensemble model that combined RF, Naïve Bayes, and Decision Tree classifiers. This stacked model attained an AUC of 0.764, substantially outperforming any single algorithm. The strong performance of this ensemble underlines the value of combining diverse computational approaches: ensemble learning methods are known to improve predictive accuracy by leveraging the complementary strengths of different algorithms.^[29–34]^ In our case, the ensemble blended a decision-tree-based learner (capturing nonlinear feature interactions) with a probabilistic classifier (handling uncertainty and feature interactions in a Bayesian framework), which likely helped generalize better in a moderate-sized, heterogeneous dataset. In contrast, models relying on linear feature selection (LASSO) or overly complex black-box approaches (neural networks) underperformed—a finding consistent with the notion that linear models struggle with complex nonlinear relationships, while neural networks risk overfitting in limited-sample settings. Collectively, these findings emphasize the importance of model selection in medical AI: a well-designed ensemble can yield robust predictions even where single-model approaches falter.

Beyond raw performance, we placed a strong emphasis on model interpretability. Using SHAP values, we identified the global and patient-specific importance of each feature in the prediction of AKI. Consistent with clinical intuition and prior literature, PLT emerged as the most influential predictor overall. One possible explanation is that low PLT may reflect either consumptive coagulopathy or severe systemic inflammation, both of which can compromise renal perfusion or directly damage renal tubules. The SHAP dependence plot for PLT in our study showed a nonlinear pattern: AKI risk was relatively flat at higher PLT, but increased sharply once PLT dropped below a certain threshold, suggesting a tipping point phenomenon. This kind of insight could inform clinicians to be especially vigilant about renal function when managing ICH patients with significant thrombocytopenia or coagulopathy. Importantly, our SHAP analysis also highlighted substantial heterogeneity in how risk factors contribute at the individual patient level.

The SHAP clustering heatmap revealed that features such as RBC, Hb, and comorbidities like MI or hypertension had variable impacts on different patients—in some cases even opposite directions of effect. In other words, not all AKI in ICH follows the same “profile.” For one subgroup of patients, anemia-related factors (low RBC/Hb) might be the dominant contributors to AKI risk, whereas in another subgroup, cardiovascular comorbidity and hemodynamic factors might predominate. This observation resonates with the general understanding that critically ill patients are highly heterogeneous, such that even those with the same primary diagnosis can have divergent pathways to adverse outcomes. Recent machine learning work in ICU populations has similarly used clustering and SHAP to identify distinct high-risk phenotypes, acknowledging that “one-size-fits-all” risk models may obscure important subgroups. By exposing the patient-specific drivers of risk, explainable models like ours pave the way for personalized management. For example, if an ICH patient’s risk is primarily driven by anemia and inflammation, aggressive optimization of oxygen delivery and anti-inflammatory measures might be warranted; whereas if another’s risk is driven by preexisting cardiac dysfunction, careful hemodynamic monitoring and avoidance of nephrotoxic stressors would be a priority. Overall, the integration of SHAP not only lends transparency to our model’s predictions but also enriches our biological understanding by suggesting that AKI in this context may have multiple mechanistic subtypes. This underscores a key message: effective prediction and prevention of AKI after ICH may require tailored strategies that account for individual variability, an approach well-aligned with precision medicine principles.

One of the novel findings of our study is the identification of Hb as a significant negative mediator in the pathway from chronic hypertension to AKI in ICH patients. Mediation analysis indicated that hypertensive status is associated with lower Hb levels, which in turn are linked to higher AKI incidence. This result quantitatively supports a mechanistic narrative that has been hypothesized but seldom measured: chronic hypertension can induce changes that predispose patients to anemia, and this anemia then exacerbates renal hypoxia during critical illness, facilitating AKI. Several physiological mechanisms could explain the reduction of Hb in the setting of hypertension. Firstly, poorly controlled high blood pressure is known to increase blood viscosity and cause shear stress on erythrocytes, leading to reduced red cell deformability, aggregation (rouleaux formation), and even hemolysis.^[35–39]^ In essence, long-standing hypertension can create a hostile circulatory environment for red blood cells. Secondly, hypertension often coexists with subclinical renal impairment—even before overt chronic kidney disease develops, hypertensive patients may have decreased renal erythropoietin production.^[40–43]^ This blunted erythropoietin response leads to an anemia of chronic disease, as evidenced by studies finding hypertensives with reduced glomerular filtration (eGFR < 90 ml/min) are nearly 3 times as likely to have anemia due to inadequate erythropoiesis.

In managing critically ill hypertensive patients with ICH, our findings advocate for heightened vigilance to Hb levels—not only to guide transfusion decisions for cerebral perfusion, but also as a surrogate of renal risk. Clinicians should be aware that even mild anemia in this context could herald an increased likelihood of AKI. Conversely, maintaining hemoglobin in an optimal range might confer renal protection. Some authors have gone so far as to suggest that hypertensive patients should have regular hemoglobin checks to prevent end-organ damage, since low hemoglobin is associated with increased morbidity in hypertension. While robust clinical trial evidence is lacking, it is biologically plausible that timely correction of anemia in the acute phase could improve renal outcomes.

These insights collectively “tell the story” that ICH and AKI are inextricably linked by pathophysiological bridges—and by understanding those bridges, we can begin to intervene more effectively. Looking ahead, our findings open several avenues for future research. First, there is a need for prospective clinical studies to validate the predictors and mediators identified here. For example, a prospective cohort could test whether integrating Hb levels into an AKI risk score improves predictive performance or patient outcomes. Interventional trials might explore whether maintaining Hb above a certain threshold in hypertensive-ICH patients (through transfusion or other means) can reduce AKI incidence—essentially translating our mediation finding into a testable hypothesis. Second, further mechanistic studies (perhaps in animal models or with advanced biomarkers) are warranted to unpack the “brain-kidney” crosstalk: how exactly does a neurological insult precipitate kidney injury at a molecular level, and vice versa? Unraveling these pathways could reveal drug targets (for instance, drugs that block specific inflammatory cascades or oxidative injury in the kidney after ICH). Third, our use of explainable AI should be expanded upon; future models could incorporate dynamic data (trends in urine output, blood pressure variability, etc) and use real-time SHAP monitoring to identify when a patient’s risk profile is deteriorating, thus serving as an early warning for clinicians. Lastly, international collaboration will be key. As indicated by the diverse contributions in the literature, a concerted effort—perhaps an international registry of stroke-associated AKI—could harmonize definitions and allow for larger-scale validation of risk factors and outcomes. By pooling data, the community can refine predictive models (possibly via federated learning to address privacy) and ensure that findings are generalizable across different populations and healthcare contexts. In conclusion, this work reinforces that the kidney should not be the “forgotten organ” in intracerebral hemorrhage. Instead, vigilant monitoring and proactive management of renal risk—guided by both clinical acumen and data-driven tools—should become integral to caring for these challenging patients. Through continued research at the intersection of neurology, nephrology, and data science, we move closer to a future of truly comprehensive neurocritical care where devastating complications like AKI are anticipated and averted, ultimately improving survival and recovery for patients with ICH.

Although our study proposes an effective method for predicting AKI, there are several limitations. First, the study used a retrospective, single-database design, lacking prospective validation and multicenter data, which may affect the generalizability and applicability of the results. Second, the small sample size of AKI cases may limit the statistical power and prediction accuracy of the model. In addition, the model lacks an external validation dataset, preventing a comprehensive evaluation of its external validity. Finally, due to insufficient treatment-related information, such as fluid management and nephrotoxic medications, potential confounding factors were not fully considered, which may impact the model’s predictive performance. Future studies should incorporate multicenter data, increase sample size, and gather more treatment-related information to address these limitations.

## 6. Conclusion

This study provides preliminary insights into the clinical and mechanistic links between ICH and AKI. Through bibliometric analysis, we identified a growing body of literature highlighting the increasing recognition of AKI as a critical complication in neurocritical care. The global distribution of research efforts and the evolving focus on systemic interactions underscore the interdisciplinary importance of this field.

Leveraging the MIMIC-IV database, we developed and systematically compared 120 machine learning model combinations to predict AKI in ICH patients. The ensemble model comprising RF, Naïve Bayes, and Decision Tree demonstrated moderate discriminative performance (AUC = 0.764), illustrating the potential of hybrid approaches in complex clinical prediction tasks. SHAP analysis revealed PLT as the most influential predictor, while RBC and Hb emerged as context-sensitive features exhibiting substantial heterogeneity across individuals—highlighting the potential for personalized risk profiling.

Importantly, mediation analysis identified Hb as a significant mediator in the relationship between hypertension and AKI, offering novel mechanistic insights into the neuro-renal axis. This finding suggests that anemia may serve not only as a biomarker of renal vulnerability but also as a modifiable target for intervention in hypertensive-ICH patients.

Together, these findings emphasize the utility of interpretable machine learning for early AKI risk stratification. However, it is crucial to note that these insights are preliminary, requiring external validation in larger, multicenter studies. Future research should also focus on incorporating hemodynamic parameters and testing the model’s real-time application in clinical settings to better translate these findings into individualized management pathways for improving outcomes in this high-risk patient population.

## Acknowledgments

We express our gratitude to all MIMIC participants and staff.

## Author contributions

**Conceptualization**: Junzhang Huang, Ningkun Xiao.

**Data curation**: Junzhang Huang, Ningkun Xiao.

**Validation**: Junzhang Huang.

**Visualization**: Junzhang Huang, Ningkun Xiao.

**Writing – original draft**: Junzhang Huang.

**Writing – review & editing**: Ningkun Xiao.
